# Sex determines cardiac and renal Vulnerability to chronic hemoglobin toxicity

**DOI:** 10.1016/j.biopha.2026.119439

**Published:** 2026-04-27

**Authors:** Daniela Lucas, Carlos Munoz, Andre F. Palmer, Pedro Cabrales

**Affiliations:** a Shu Chien-Gene Lay Department of Bioengineering - University of California, San Diego, CA, United States; b William G. Lowrie Department of Chemical and Biomolecular Engineering, The Ohio State University, Columbus, OH, United States

**Keywords:** Hemoglobin toxicity, Hemolytic anemia, Oxidative stress, Mitochondrial dysfunction, Bioenergetics, Cardiac injury, Renal injury, Sex differences

## Abstract

Hemolytic anemias are characterized by chronic intravascular hemolysis, leading to sustained release of acellular hemoglobin (Hb). Circulating Hb scavenges nitric oxide, releases redox-active heme, and promotes oxidative and inflammatory injury, contributing to progressive cardiac and renal dysfunction. Despite the known cardiopulmonary and renal complications in hemolytic disorders, the direct mechanisms of Hb-mediated injury and the influence of sex as a biological variable remain partially understood. In this study, we investigated the effects of chronic Hb exposure on cardiac and renal function in male and female mice. Animals received daily intraperitoneal injections of acellular Hb (320 mg/kg) for six weeks. Cardiac function was evaluated by echocardiography, renal function by transdermal glomerular filtration rate, and tissue injury biomarkers were quantified by ELISA. Mitochondrial respiratory function was also assessed in cardiac and renal tissues. Chronic Hb exposure induced significant cardiac dysfunction and renal impairment in male mice, evidenced by reduced ejection fraction, decreased stroke volume, diminished glomerular filtration rate, and elevated injury biomarkers. In contrast, female mice showed less impairment of cardiac and renal function and exhibited lower levels of biomarkers. Mitochondrial respiration revealed marked reductions in bioenergetics in males, whereas females maintained superior mitochondrial bioenergetics in both organs. These findings identify mitochondrial dysfunction as a mechanistic link between chronic Hb exposure and multi-organ injury and demonstrate sex-dependent bioenergetic adaptability as a key determinant of disease severity. Collectively, this work underscores the importance of incorporating sex as a biological variable and supports the development of Hb-neutralizing therapeutic strategies to mitigate Hb-induced organ damage.

## 1. Introduction

Chronic hemolysis is a common occurrence in hematological disorders, including sickle cell anemia, alpha and beta thalassemia, spherocytosis, and hemochromatosis [1–5]. While patients with these conditions often experience multi-organ complications over time, the role of circulating free hemoglobin (Hb) toxicity as an early driver of downstream pathology remains unclear. Hemolysis is the breakdown of red blood cells (RBCs) in which their internal components are released into the bloodstream, including primarily Hb, cell fragments, and bilirubin [6–10]. In the context of blood disorders, most hemolysis arises from structurally abnormal RBCs, limiting their deformability to travel though the microcirculation or from unstable Hb variants that precipitate within the RBCs, compromising membrane integrity and triggering lysis [1–3].

Although chronic hemolysis is associated with progressive renal dysfunction, adverse cardiac remodeling, and pulmonary hypertension, these downstream events are often attributed to disease progression rather than to direct organ Hb toxicity [11–14]. However, evidence from Hb-based oxygen carrier (HBOC) clinical trials and preclinical studies has demonstrated the correlation between plasma Hb levels and organ injury, particularly in the heart and kidneys [15–18]. Free Hb rapidly scavenges nitric oxide (NO), impairing vasodilation, reducing oxygen delivery, and promoting vasoconstriction [19–21]. Furthermore, once released from RBCs, Hb becomes unstable, dissociating into dimers and releasing its heme group and iron [6,22]. These smaller molecules can extravasate into the extravascular space, inducing oxidative stress, inflammation, and ultimately leading to cellular injury or death [23–26]. These mechanisms underscore the importance of isolating the direct effects of chronic Hb exposure from confounding factors associated with hemolytic anemias. These include associated inflammation, compensatory erythropoietic responses, and the influence of other erythrocyte-derived components released during hemolysis [including cell membrane fragments, lactate dehydrogenase, arginase-1, adenosine diphosphate (ADP), adenosine triphosphate (ATP), potassium, free iron, and heme] [8,10].

Growing evidence suggests that among the population diagnosed with hemolytic anemias, females have more favorable outcomes compared to males [27,28]. For example, past research has shown that females with sickle cell disease tend to maintain renal function more effectively over time. In contrast, males often exhibit a more rapid decline in kidney performance [27]. Additionally, when transfusions are required, females have been shown to achieve greater improvements in oxygen delivery compared to males [27]. Beyond hemolytic disorders, females also demonstrate slower progression of chronic kidney disease and a lower incidence of cardiovascular mortality and heart failure [29–33]. These observations highlight the importance of considering sex as a biological variable in understanding the systemic impact of chronic hemolysis. Even with evidence suggesting sex disparities in outcomes, the mechanisms underlying Hb toxicity and possible sex disparities in chronic settings remain poorly understood.

This study aims to elucidate the impact of chronic Hb toxicity by evaluating functional, molecular, and energetic parameters for renal and cardiac systems in healthy animals after six weeks of daily Hb injections. Cardiac function was assessed via echocardiograms, while kidney function was measured via transdermal GFR. In addition, systemic, cardiac, and renal markers of function and inflammation were analyzed, coupled with mitochondrial energetic respiration. Identifying the contribution of chronic Hb toxicity to organ injury and how it varies between males and females may offer critical insight into disease progression and inform sex-specific therapeutic interventions.

## 2. Methods

### 2.1. Solution preparation

The human Hb used in these studies was purified from human RBCs via tangential flow filtration as described in the literature [34]. Briefly, the Hb was purified through a multistage tangential flow filtration (TFF) process using sequential 50 nm, 500 kDa, 100 kDa, and 50 kDa hollow fiber membranes to remove impurities from the RBC lysate. The purity of the Hb was confirmed via electrophoresis. Gel was stained with Coomassie blue R250 (stain buffer, Bio-Rad) for 1 h and then destained with destaining buffer (10% acetic acid and 20% methanol). The gels were scanned on a Gel Doc XR (Bio-Rad) imaging system and analyzed with the Quantity One 1-D analysis software (Bio-Rad Laboratories, Hercules, CA). Endotoxin level of purified Hb was measured using an endotoxin test kit (Pyrogent Plus, Lonza, Walkersville, MD).

### 2.2. Chronic Hb dosing

Male and female C57BL/6 J mice were dosed daily for 6 weeks with acellular Hb solution. Before each injection, animals were briefly anesthetized with isoflurane (3% /vol, Dragerwerk AG, Lübeck, Germany) ¨ and received intraperitoneal injections. The Hb solution was administered at a concentration of 10 g/dL, corresponding to 5% of the animal's blood volume, calculated based on body weight. The Hb dose was selected based on the rate of destruction of RBCs observed in severe hemolytic disorders [35]. While healthy individuals replace roughly 1% of their RBCs daily, those with hemolytic anemia experience accelerated destruction that can range up to 3%.

### 2.3. Echocardiograms

Two-dimensional and M-mode transthoracic echocardiography was performed using a Vevo 2100 Microimaging system (VisualSonics) equipped with a 40-MHz high-frequency transducer. The imaging platform was maintained at a physiological temperature (37°C) to prevent hypothermia and sickling in the mice. Mice were anesthetized with 1.5% isoflurane, and heart rate and core body temperature were continuously monitored throughout the procedure. Cardiac chamber dimensions and ventricular function were assessed using two-dimensional-guided M-mode images acquired from the parasternal short-axis view. Doppler images were obtained from the apical four-chamber view following established consensus guidelines [36,37]. Annular dimensions were measured in the parasternal long-axis view during systole for semilunar valves, and in the apical four-chamber view during diastole for atrioventricular valves. All measurements were obtained using a consistent angle of interrogation, recorded in triplicate, and averaged. Ultrasound images were stored on the system’s hard drive and subsequently transferred to a secure image server for offline analysis. Each echocardiographic session lasted approximately 20 min per animal, and all measurements were performed at the same time of day to minimize the effects of circadian variability.

### 2.4. Animal preparation

At the termination of 6-week chronic dosing, experiments were conducted using 13-week-old male and female C57BL/6 J mice (Jackson Laboratories, ME) with weights ranging from 20 to 25 g. Animal care and handling adhered to the NIH Guide for the Care and Use of Laboratory Animals, and the experimental protocol was approved by the UCSD Institutional Animal Care and Use Committee. Animals were anesthetized with isoflurane (5%/vol for induction, 2.5%/vol for maintenance, Drägerwerk AG, Lübeck, Germany) and underwent carotid artery catheterization. The animals were positioned supine on a heating pad to maintain their core body temperature at 37°C and were allowed to breathe freely from a nosecone. After surgery, the isoflurane concentration was reduced to 1.0%/vol. Anesthesia depth was continually monitored using a toe pinch, and the isoflurane concentration was adjusted by 0.1%/vol if necessary to ensure the animal's comfort

### 2.5. Kidney function

The day before GFR measurements, the fur on the back of the animals was removed using Nair. On the day of the procedure, anesthetized mice were placed in a prone position on a heated pad for device placement. The transdermal GFR monitoring device (MediBeacon, St. Louis, MO) was placed on one side of the adhesive patch, ensuring that the LEDs were positioned directly over the clear window. The battery was connected to the device, and once the red LED began blinking, data acquisition started. The device was securely attached to the shaved skin, with the LED window positioned over the ribcage. The blinking LED turned green upon successful skin detection. To minimize light interference, the animal was covered with aluminum foil. The device was left in place for 5 min before FITC-sinistrin injection to establish a baseline signal. Following the 5-minute stabilization period, and after the device ceased blinking red, FITC-sinistrin was prepared in a 1 mL syringe at a dose of 70 mg/kg (diluted 28 mg of FITC-Sinistrin Medibeacon in 1 mL of sterile saline), rounded to the nearest 10 μL. The solution was administered via the femoral catheter placed during surgery. After injection, the catheter was flushed with saline to ensure complete circulation of the FITC-sinistrin. GFR measurements were recorded over a 1-hour period, after which the device was removed.

### 2.6. Systemic hemodynamics parameters

Mean arterial pressure (MAP) and heart rate (HR) were recorded continuously from the carotid artery using a PowerLab 8/30 data acquisition system and analyzed with LabChart 7 software (ADInstruments, Colorado Springs, CO). Arterial blood was collected at the end of the experiment in heparinized glass capillary tubes (65 μL) and immediately analyzed for blood chemistry and gases (ABL90; Radiometer America, Brea, CA).

### 2.7. Markers of organ damage and inflammation

After the experimental protocol, the organs were harvested for molecular marker and histological analysis. Kidney injury molecule-1 (KIM-1) was measured in kidney tissue homogenates using an ELISA kit (ERHAVCR1, Thermo Fisher, Waltham, MA). Kidney and plasma cytokine levels of interleukin-6 (IL-6) and interleukin-10 (IL-10) were quantified using ELISA kits (BMS625 and BMS629, respectively, Thermo Fisher). Urinary creatinine was measured using kit EIACUN (Thermo Fisher). Neutrophil gelatinase-associated lipocalin (uNGAL) and albumin were measured using kits ERLCN2 and EEL124, respectively (Thermo Fisher). Plasma cardiac troponin I was quantified using an ELISA kit (EEL112, Thermo Fisher). Liver function was measured from plasma bilirubin using kit NBP2–69939 (Novus Biologicals), and aspartate transaminase (AST) using kit EEL086 (Thermo Fisher). Blood urea nitrogen (BUN) was measured using kit 0544-MBS2611086–96 (Biomeda Corp). Urinary iron was assessed using kit ab83366, and plasma heme was measured using kit ab272534 (Abcam).

### 2.8. Mitochondrial respiration

Mitochondrial respiration was assessed using a Seahorse XFe96 Analyzer (Agilent Corp) on isolated mitochondria. Cardiac and renal tissue were homogenized in ice-cold isolation buffer (e.g., 250 mM sucrose, 10 mM HEPES, 1 mM EGTA, pH 7.4) using a glass-Teflon homogenizer, followed by differential centrifugation to pellet mitochondria. mitochondrial assay buffer (MAS) consisting of 70 mM sucrose, 220 mM mannitol, 10 mM KH_2_PO_4_, 5 mM MgCl₂, 2 mM HEPES, 1 mM EGTA, and 0.2% (w/v) BSA, adjusted to pH 7.2 at 37 °C. Mitochondrial protein concentration was determined by BCA assay for normalization. An equal mass of mitochondria (e.g., 5–10 μg protein/well) was seeded per well of the XF cell culture microplate, centrifuged briefly to adhere mitochondria to the bottom of the well, and incubated at 37 °C in a non-CO_2_ incubator for 10–15 min before measurement. Prior to the assay, culture medium was exchanged for Seahorse XF DMEM Basal Medium supplemented with 2 mM glutamine, 10 mM glucose, and 1 mM sodium pyruvate, prepared 2 h before the assay and maintained for the duration of the measurement. Oxygen consumption rate (OCR) was measured at baseline and after sequential injections of mitochondrial inhibitors administered during the measurements as follows: oligomycin (2.5 μM; ATP synthase inhibitor), FCCP (2 μM; uncoupler), and a combination of rotenone and antimycin A (2.5 μM each; complex I/III inhibitors) to quantify basal, maximal, and non-mitochondrial respiration, respectively. Data were normalized to mitochondrial mass (protein content) to allow comparison between groups.

### 2.9. Mitochondrial ROS

ROS production in heart and kidney mitochondria was evaluated using the 2 ′,7 ′-dichlorofluorescein diacetate (DCFH2-DA) staining assay (Thermo Fisher Scientific). Briefly, incubated with DCFH2-DA for 30 min at 37°C in the dark, washed, resuspended in PBS, and maintained on ice for immediate assay by flow cytometry (BD Biosciences). To mitigate probe artifacts, positive controls (antimycin A) and negative controls (FCCP) were included on every plate, and readings were taken within the linear range. The data were analyzed using FACSDiva software (BD) to calculate the fluorescent intensity.

### 2.10. Lipid peroxidation analysis

Lipid peroxidation was quantified in plasma and renal tissue homogenates using commercial colorimetric and ELISA-based assays (Abcam, Cambridge, UK: ab287803, ab65622, ab233471). Tissues were homogenized in ice-cold PBS containing 0.05% butylated hydroxytoluene (BHT) and centrifuged at 10,000 × g for 10 min at 4 °C; supernatants were analyzed immediately. Malondialdehyde (MDA) was measured using the colorimetric assay (ab233471) and expressed as nmol MDA per mg protein. 4-Hydroxynonenal (4-HNE) was quantified by competitive ELISA (ab287803) and expressed as pg per mg protein. Phosphate content was determined for quality control using kit ab65622 and did not affect peroxidation results. All samples and standards were run in duplicate, background absorbance was subtracted, and values were normalized to total protein (BCA).

### 2.11. Statistical analysis

Each experimental group consisted of 8–9 animals. Data are presented as individual values. All statistical analyses were performed using two-way ANOVA to assess the main effects of treatment and sex, as well as their interaction, followed by Tukey’s HSD post hoc test for multiple comparisons. A p < 0.05 was considered statistically significant. All analyses were conducted using GraphPad Prism 10 (GraphPad Software, San Diego, CA, USA).

## 3. Results

**Fig. 1.**
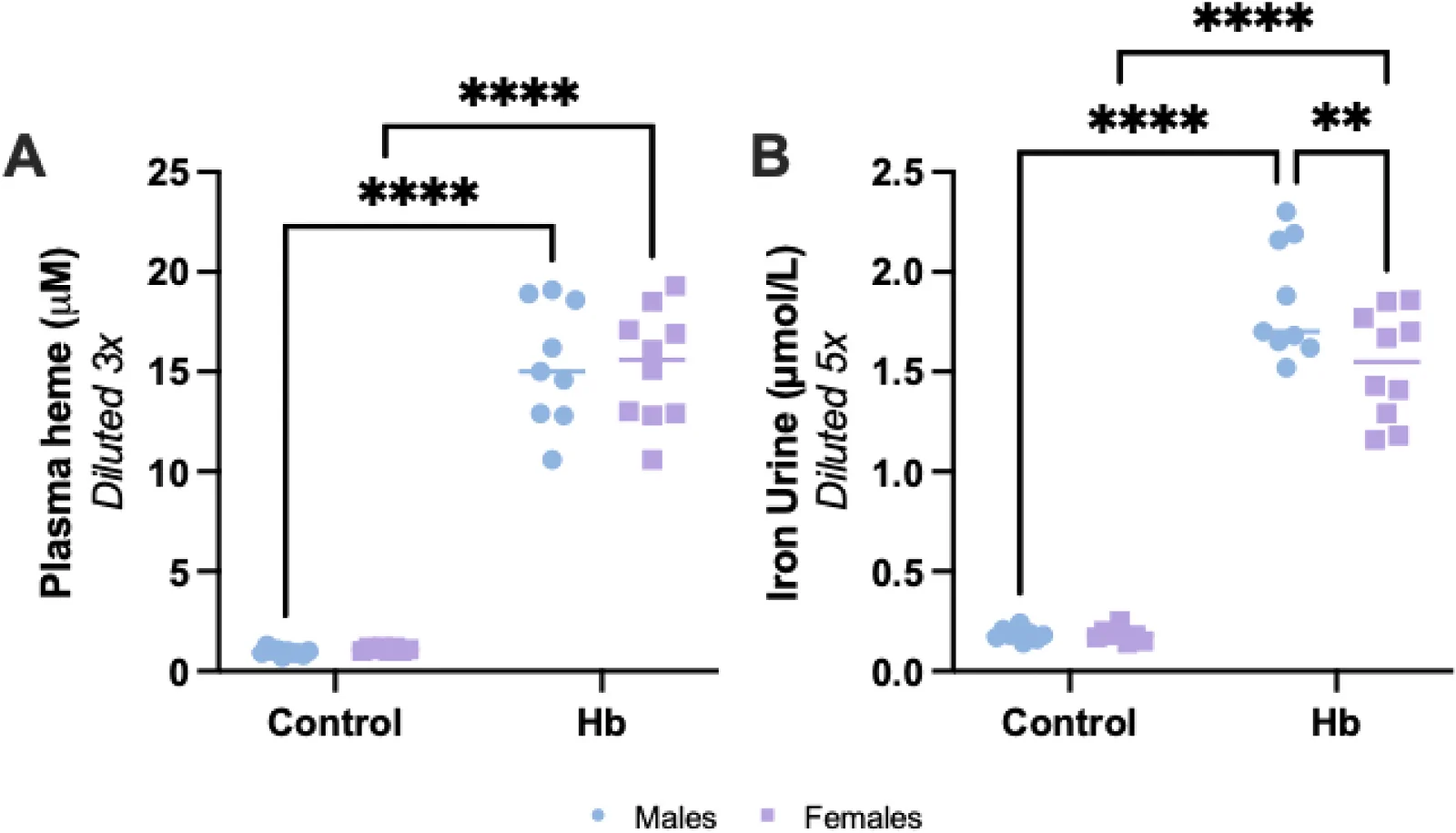
Plasma Heme and Urine Iron After Hemoglobin Exposure. Plasma heme levels were significantly elevated in both Hb-exposed females and males, indicating increased circulating heme due to Hb in broth groups. Urinary iron levels showed a significant decrease in females compared to males, though no significant differences were in plasma heme. Data is presented as individual values. Statistical significance between groups is denoted by * (p < 0.05), **(p < 0.001), and *** (p < 0.0001).

To establish the chronic Hb exposure model, we first confirmed elevated circulating heme levels following daily Hb administration (Fig. 1). Hb-treated animals exhibited increased plasma heme compared to controls, verifying systemic exposure and persistence of circulating Hb throughout the study period. No significant differences were observed between sexes, indicating comparable heme exposure in males and females. However, urinary heme-to-iron deposition was higher in males than in females, although both groups were significantly elevated relative to controls.

Body weight differed between sexes, with males (Control: 28.8 ± 1.4 g and Hb: 26.7 ± 2.8 g) weighing more than females (Control: 20.4 ± 1.4 g and Hb: 19.8 ± 1.0 g). Because no significant differences were observed between treatment groups within each sex, functional parameters were normalized to their respective controls as ratios, allowing for accurate comparison of Hb-induced changes relative to each sex’s baseline.

### 3.1. Cardiac functional and molecular assessment

**Fig. 2.**
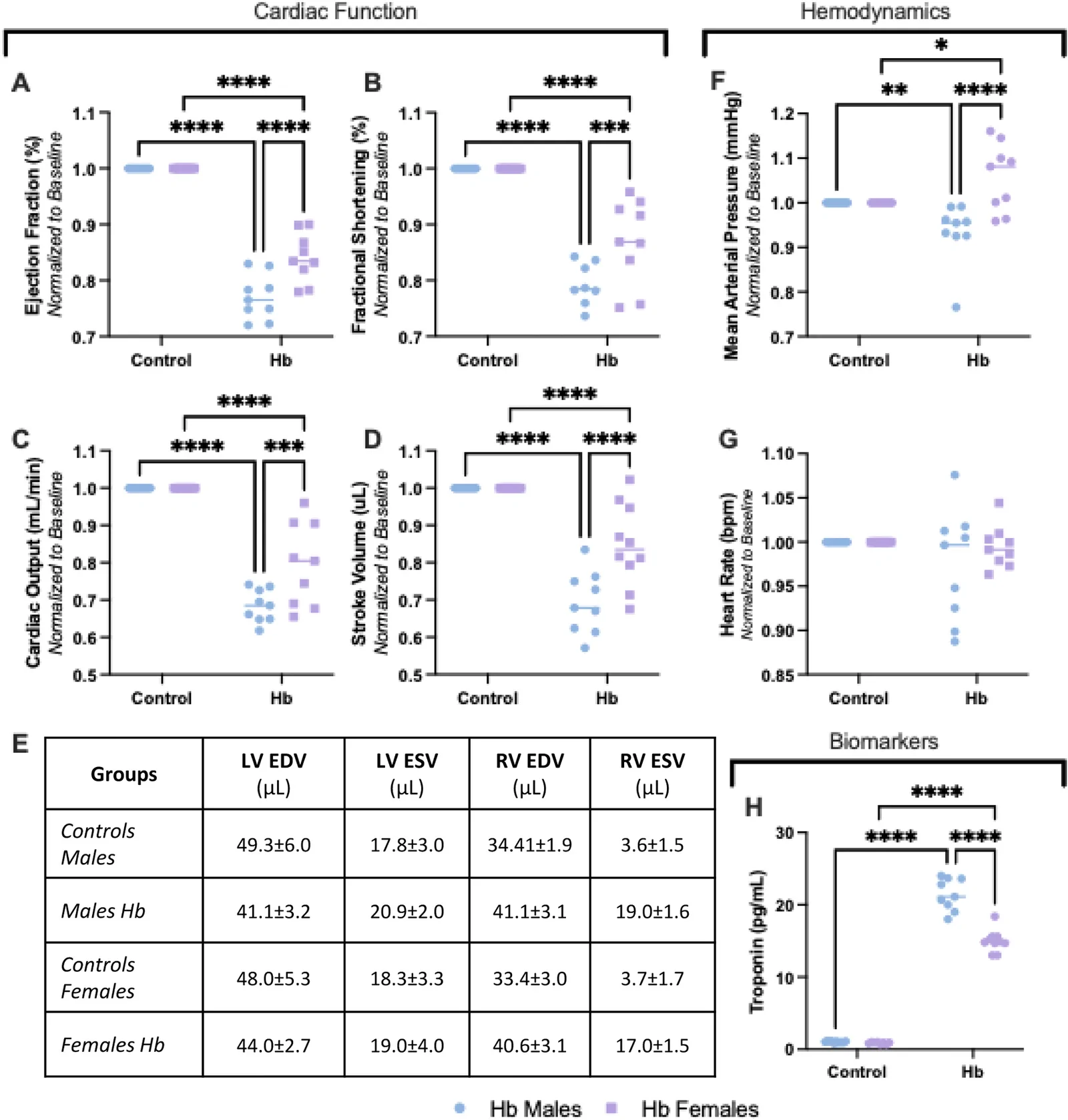
Cardiac Functional and Molecular Assessment Six Weeks Post-Chronic Hemoglobin (Hb) Infusions. Echocardiography demonstrated a significant reduction in cardiac function in males compared with females following six weeks of chronic Hb exposure. (A–D) Functional parameters, including (A) ejection fraction, (B) fractional shortening, (C) cardiac output, and (D) stroke volume, are presented as ratios normalized to sex-matched controls and show that females maintain higher cardiac performance than males during chronic Hb exposure. (E) The table summarizes raw ventricular volumes, showing comparable baseline values between control males and females, confirming similar starting cardiac function. (F–G)Hemodynamic measurements normalized to baseline, (F) mean arterial pressure, and (G) heart rate, were significantly impaired in males compared with females. (H) Cardiac troponin, a biomarker of myocardial injury was significantly elevated in males compared to females, consistent with greater male susceptibility to Hb-induced cardiotoxicity. Data are presented as individual values. Statistical significance between groups is denoted by * (p < 0.05), **(p < 0.001), and *** (p < 0.0001).

Cardiac functional measurements, hemodynamics, and biomarkers are shown in Fig. 2. As illustrated, male animals exhibited a significant reduction in cardiac function compared to females. Left ventricular ejection fraction (EF) and fractional shortening (FS) were significantly decreased in Hb-treated males relative to females (Fig. 2A–B), suggesting that chronic Hb exposure exerts cardiotoxic effects, likely through extravasation into the myocardium. This impairment could arise from disrupted calcium handling or tissue injury via oxidative stress in myocardial tissue, both of which are known to compromise cardiac contractility. Consistent with these reductions in EF and FS, stroke volume (SV) and cardiac output (CO) were also significantly lower in males than females (Fig. 2C–D), reflecting diminished overall cardiac performance under chronic Hb exposure. Ventricular volumes left ventricular (LV) end diastolic volume (EDV), LV end systolic volume (ESV), right ventricular (RV) EDV, and RV ESV, were comparable between control males and females (Fig. 2E), confirming similar baseline cardiac structure and indicating that the observed sex differences result from Hb-induced dysfunction rather than pre-existing disparities.

After six weeks of Hb dosing, hemodynamics were measured via a carotid catheter and monitored throughout the 60 min during GFR assessment. Both mean arterial pressure (MAP) and heart rate (HR) were significantly higher in females compared with males (Fig. 2F–G), indicating that females were able to maintain cardiovascular stability under chronic Hb exposure. Relative to their respective controls, males showed significant deviations in both MAP and HR, whereas females only changed in MAP, indicating that females were able to maintain function and energy to support HR under chronic Hb challenge. In addition to functional impairments, cardiac troponin levels (Fig. 2H), a biomarker of cardiomyocyte injury, was elevated in males relative to females, further indicating increased susceptibility of male hearts to chronic Hb toxicity. Nonetheless, both groups were elevated compared to their controls due to Hb exposure.

Lastly, qualitative data from histological stains of the myocardium with picrosirius red show an increase in collagen deposition in the Hb groups compared to their controls, suggesting that Hb-induced cardiac dysfunction is accompanied by fibrosis and structural remodeling (see Supplemental Figure 1). These findings indicate that female mice are relatively protected from the toxic effects of chronic Hb exposure, maintaining both structural and functional cardiac integrity despite prolonged Hb exposure, while males exhibit a functional decline and an increase in myocardial injury and fibrosis.

### 3.2. Hb exposure impairs cardiac mitochondrial respiration in a sex-dependent manner

**Fig. 3.**
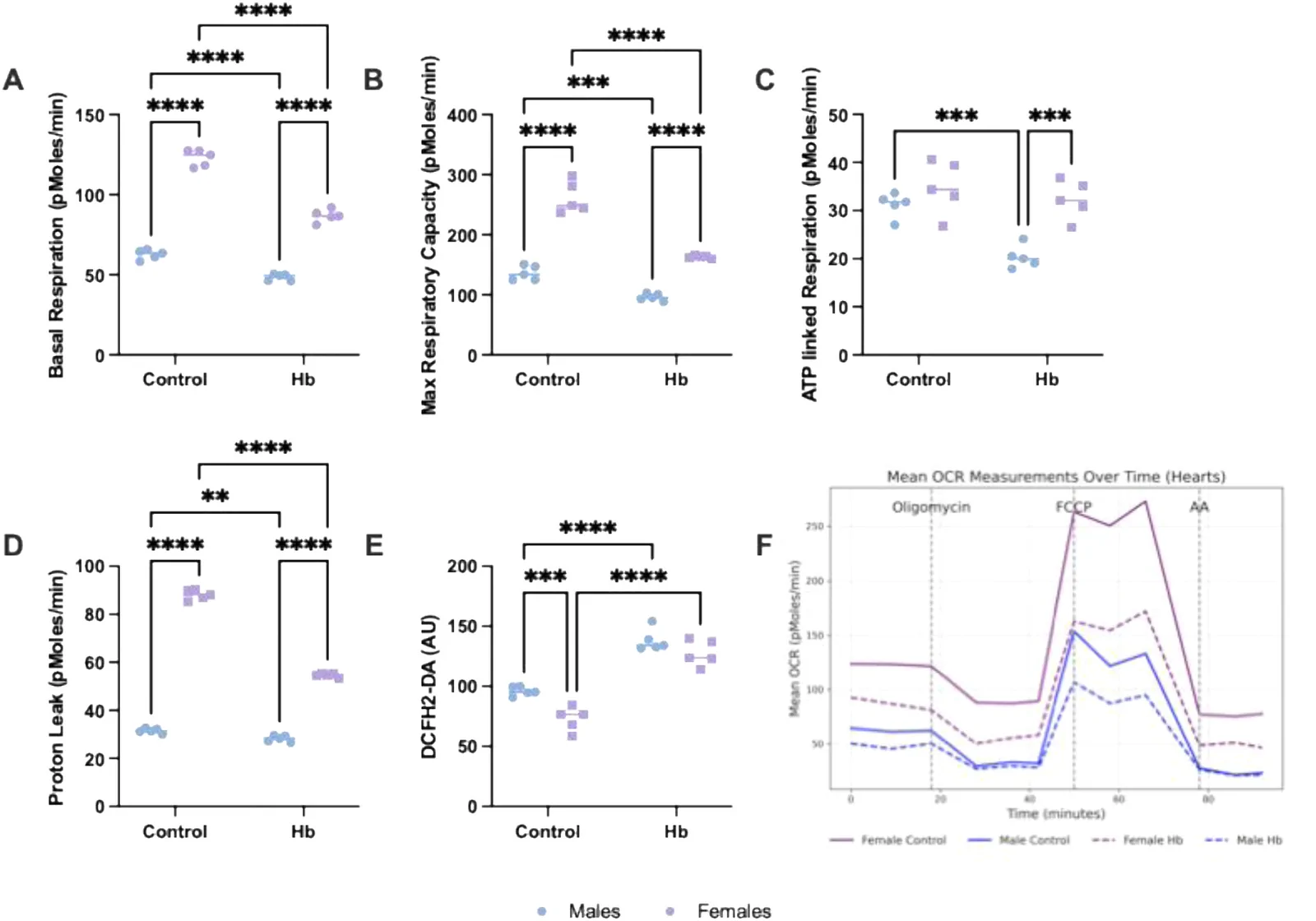
Mitochondrial Respiration in Cardiac Tissue Following Chronic Hemoglobin (Hb) Infusions. Mitochondrial respiration was assessed using high-resolution respirometry after six weeks of chronic Hb exposure. (A–F) Quantification of key mitochondrial parameters demonstrates significant reductions in (A) basal respiration, (B) ATP-linked respiration, (C) maximal respiratory capacity, (D) and proton leak in males compared with females, indicating different mitochondrial bioenergetics. (E) Reactive Oxygen Species show no significant sex differences but indicate a lower trend in females. (F) Representative oxygen consumption rate (OCR) traces illustrating all experimental groups, from which mitochondrial respiration parameters were derived. Sequential injections of oligomycin (ATP synthase inhibitor), FCCP (uncoupler), and rotenone/antimycin A (complex I/III inhibitors) were used to quantify basal, ATP-linked, proton leak, and maximal respiration. Data is presented as individual values. Statistical significance between experimental groups is denoted by * (p < 0.05), **(p < 0.001), and *** (p < 0.0001.

Chronic Hb exposure led to significantly altered mitochondrial profiles in male cardiac tissue compared with females, as shown in Fig. 3. Basal respiration was significantly reduced in males, indicating a lower mitochondrial oxygen consumption under resting conditions (Fig. 3A). ATP-linked respiration was significantly decreased, suggesting impaired oxidative phosphorylation and reduced ability to meet energetic demands (Fig. 3B). Maximal respiratory capacity was diminished in males, demonstrating a limited capacity for male mitochondria to respond to metabolic stress (Fig. 3C). Moreover, females also showed a significantly higher proton leak compared to males (Fig. 3D). Reactive oxygen species (ROS) production did not differ significantly between sexes, although females showed a trend toward lower ROS generation (Fig. 3E). Finally, an OCR trace for each experimental group (Fig. 3F), illustrating group-specific differences in mitochondrial bioenergetics under the addition of inhibitors and uncouplers used to assess basal, ATP-linked, proton leak, and maximal respiration.

It is important to note that both sexes showed declines relative to their respective controls, indicating that chronic Hb exposure impairs cardiac mitochondrial energetics. The observed mitochondrial changes in males corresponded with increased cardiac troponin levels, indicating ongoing cardiomyocyte injury. Impaired ATP production and reduced mitochondrial reserve likely limit the heart’s ability to maintain contractile function and cellular homeostasis under stress, rendering cardiomyocytes more susceptible to apoptosis and injury. Notably, females maintained ATP-linked respiration at levels comparable to their baseline controls, indicating preserved oxidative phosphorylation despite chronic Hb exposure. This lack of deviation from baseline shows preserved energy production capacity, which likely contributes to the higher cardiac performance and reduced injury biomarkers observed in females. These findings support a mechanistic link between chronic Hb-induced bioenergetic deficits and myocardial injury, as reflected by damage to heart muscle cells, which leaks troponin into the bloodstream.

### 3.3. Renal functional and molecular assessment

**Fig. 4.**
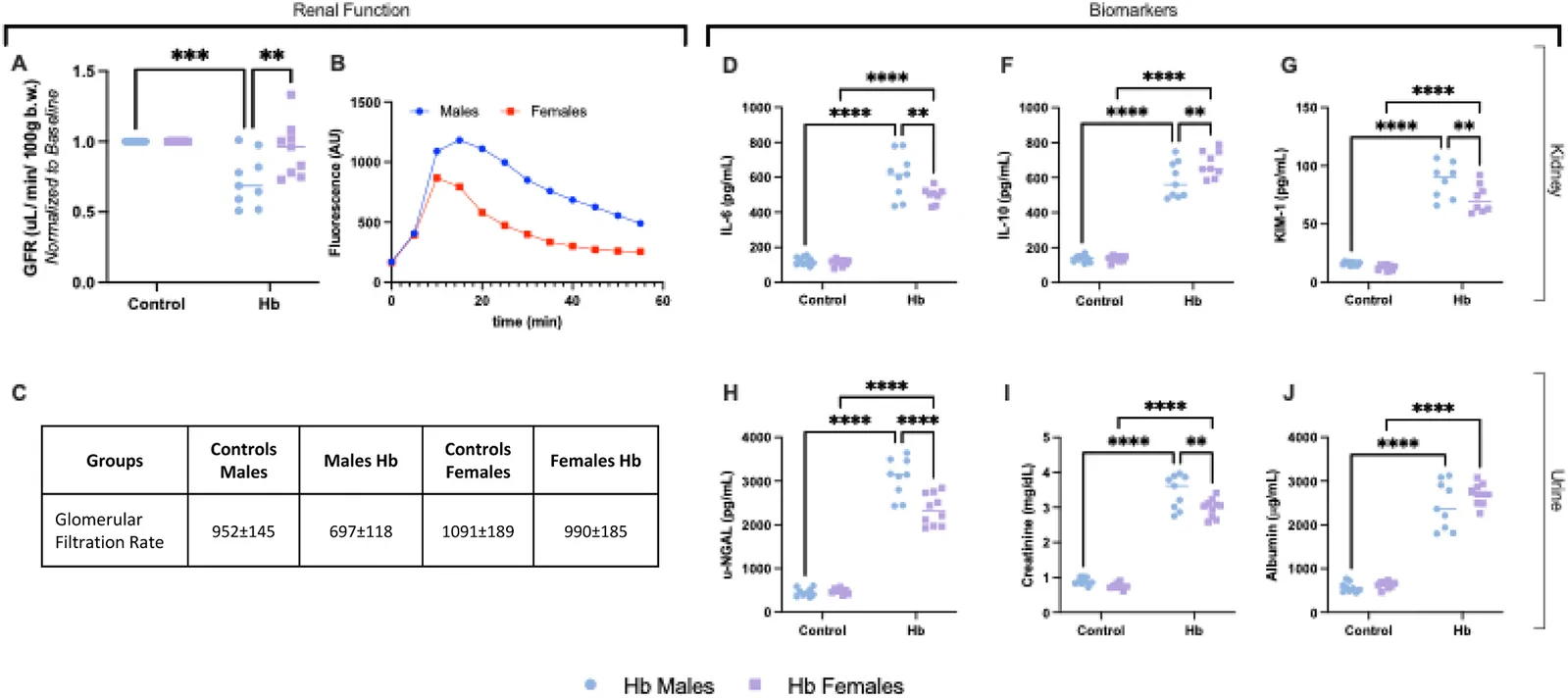
Renal Functional and Molecular Assessment Six Weeks Post Chronic Hemoglobin (Hb) Infusions. Female m1ice are protected from hemoglobin (Hb)–induced renal dysfunction. (A) Glomerular filtration rate (GFR) normalized to sex-matched controls shows a significant reduction in Hb-treated males compared to controls and females. (B) Fluorescence clearance curves demonstrate delayed renal clearance in males relative to females after Hb exposure. (C) Table shows group averages ± SD for absolute GFR values, confirming comparable baseline renal function in male and female controls (D–J) Biomarkers of kidney injury reveal sex-specific differences: (D) IL-6 levels are elevated in males, while (F) IL-10 is higher in females, consistent with anti-inflammatory protection. (G) Kidney injury molecule-1 (KIM-1), (H) urinary NGAL (u-NGAL), and (I) creatinine are significantly higher in males, indicating greater renal injury, whereas females show relative preservation. (J) Albumin excretion trends higher in males but does not reach significance. Data is presented as individual values. Statistical significance between groups is denoted by * (p < 0.05), **(p < 0.001), and *** (p < 0.0001).

Renal function and injury biomarkers revealed sex-specific differences following chronic Hb exposure, as shown in Fig. 4. Transdermal glomerular filtration rate (GFR) was significantly reduced in Hb-treated males compared to male controls, whereas females exhibited a smaller reduction in GFR despite chronic Hb exposure. Moreover, an example of fluorescence clearance curves (Fig. 4B) for both males and females demonstrated delayed clearance kinetics in males, while females exhibited faster elimination, suggesting more efficient renal handling of circulating Hb. The table presented in panel C shows the absolute GFR values (means ± SD) for each group. Moreover, molecular biomarkers (Fig. 4D–J) measured in the kidney and urine indicated different responses between sexes, with lower injury and inflammatory biomarkers in females compared to males. IL-6, a pro-inflammatory cytokine, was significantly elevated in males compared to females, whereas IL-10, an anti-inflammatory cytokine, was higher in females, suggesting that females have a more protective immunomodulatory response to Hb stress. Additionally, markers of tubular injury also supported this trend. Hb-treated males displayed elevated KIM-1, urine creatinine, and u-NGAL levels compared to females, consistent with greater tubular injury and impaired renal function. All markers, regardless of sex, were elevated compared to the controls, showing the toxic effect of Hb. Together, these results indicate that chronic Hb exposure leads to renal dysfunction, and specifically tubular injury. Males appear to be more sensitive to stress induced by Hb, while females exhibit relative preservation of filtration capacity and reduced injury, likely due to a combination of sex dependency and protective anti-inflammatory signaling.

### 3.4. Hb exposure impairs renal mitochondrial respiration in a sex-dependent manner

**Fig. 5.**
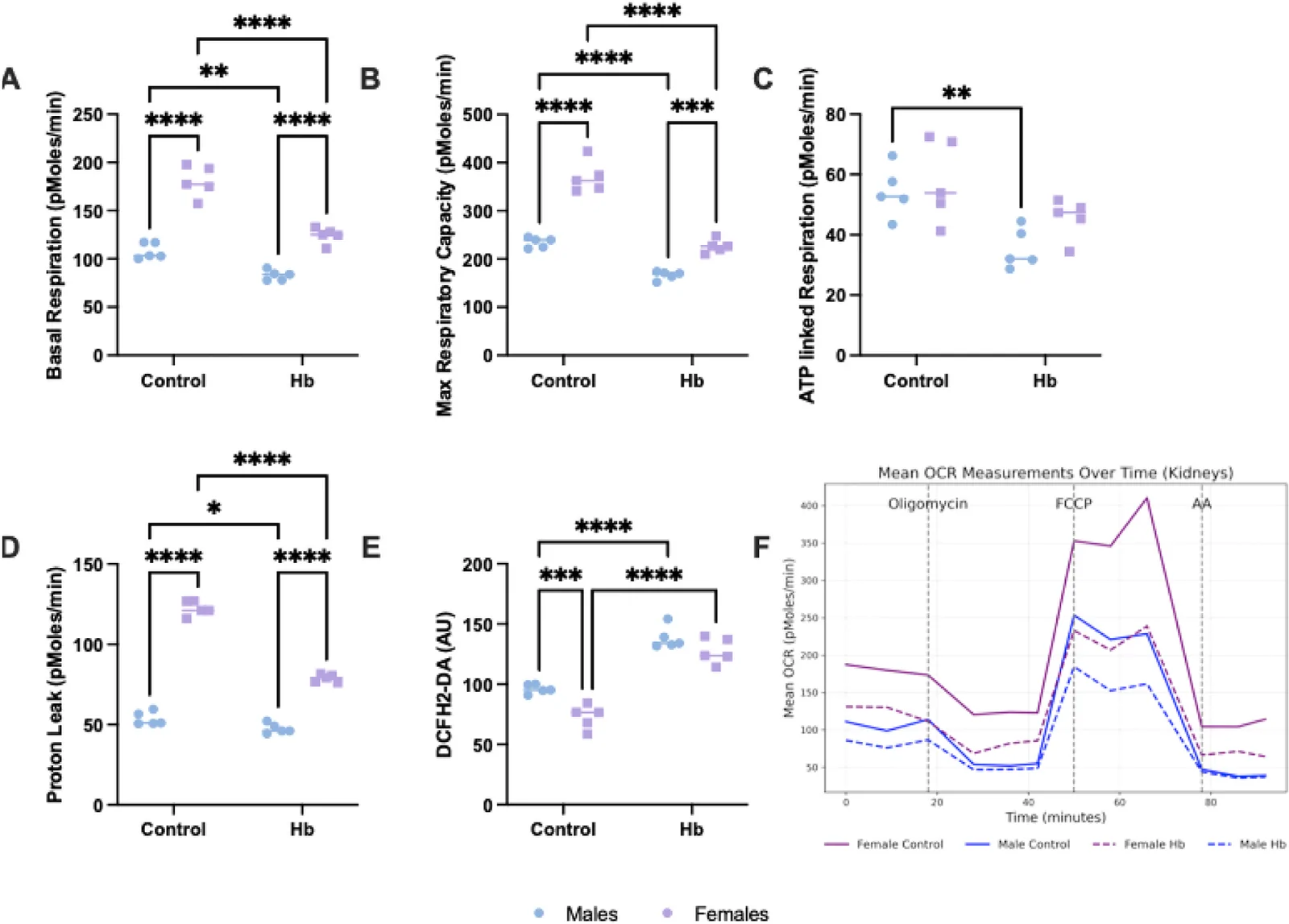
Mitochondrial Respiration in Renal Tissue Following Chronic Hemoglobin (Hb) Infusions. Mitochondrial respiration was assessed using high-resolution respirometry after six weeks of chronic Hb exposure. (A–F) Quantification of key mitochondrial parameters demonstrates reductions in (A) basal respiration, (B) ATP-linked respiration, (C) maximal respiratory capacity, (D) proton leak in males compared with females, indicating impaired mitochondrial bioenergetics. (E) Reactive Oxygen Species show no significant sex differences in Hb groups, but it indicates a lower trend in females. (F) Representative oxygen consumption rate (OCR) traces illustrating all experimental groups, from which mitochondrial respiration parameters were derived. Sequential injections of oligomycin (ATP synthase inhibitor), FCCP (uncoupler), and rotenone/antimycin A (complex I/III inhibitors) were used to quantify basal, ATP-linked, proton leak, and maximal respiration. Data are presented as individual values. Statistical significance between experimental groups is denoted by * (p < 0.05), **(p < 0.001), and *** (p < 0.0001).

Chronic Hb exposure led to significantly altered mitochondrial profiles in male renal tissue compared with females, as shown in Fig. 5. Basal respiration was significantly reduced in males (Fig. 5A), and ATP-linked respiration was significantly decreased, suggesting impaired oxidative phosphorylation and reduced ability to meet energetic demands (Fig. 5B). Maximal respiratory capacity was diminished in males, demonstrating a limited capacity for male mitochondria to respond to metabolic stress (Fig. 5C). Moreover, females also showed slightly higher proton leak compared to males (Fig. 5D). The elevated proton leak observed in females likely represents mild mitochondrial uncoupling that limits electron transport chain over-reduction, thereby reducing ROS generation and protecting against oxidative injury [38–40]. Controlled uncoupling of this type has been shown to preserve mitochondrial integrity and enhance cellular resilience under metabolic stress [38–40]. By contrast, males displayed lower respiration and lower proton leak, consistent with impaired mitochondrial capacity and a reduced ability to buffer against metabolic stress. ROS (Fig. 5E) trended lower in females but did not show significant differences between sexes. Finally, an OCR trace for each experimental group (Fig. 5F), illustrating group-specific differences in mitochondrial bioenergetics under the addition of inhibitors and uncouplers used to assess basal, ATP-linked, proton leak, and maximal respiration.

Despite superior female mitochondrial respiration, both sexes showed declines relative to their respective controls, indicating that chronic Hb exposure impairs renal mitochondrial respiration. Interestingly, inherent mitochondrial differences were observed in control animals, with female mitochondria exhibiting greater baseline capacity, highlighting their ability to better adapt to injury. These mitochondrial deviations in males with Hb corresponded with increased renal injury and inflammatory biomarkers in both the kidney and urine, reflecting the kidney challenge. Impaired ATP production and reduced mitochondrial reserve likely limit the kidneys’ ability to sustain high filtration rates, a metabolically demanding process, making renal cells more susceptible to apoptosis and necrosis [41,42]. By contrast, females maintained ATP-linked respiration at levels comparable to their baseline controls. This preservation correlates with their maintained kidney function and lower injury biomarker levels, underscoring organ-specific, sex-dependent resilience to Hb-induced injury.

### 3.5. Lipid peroxidation indicates systemic oxidative injury following chronic Hb exposure

**Fig. 6.**
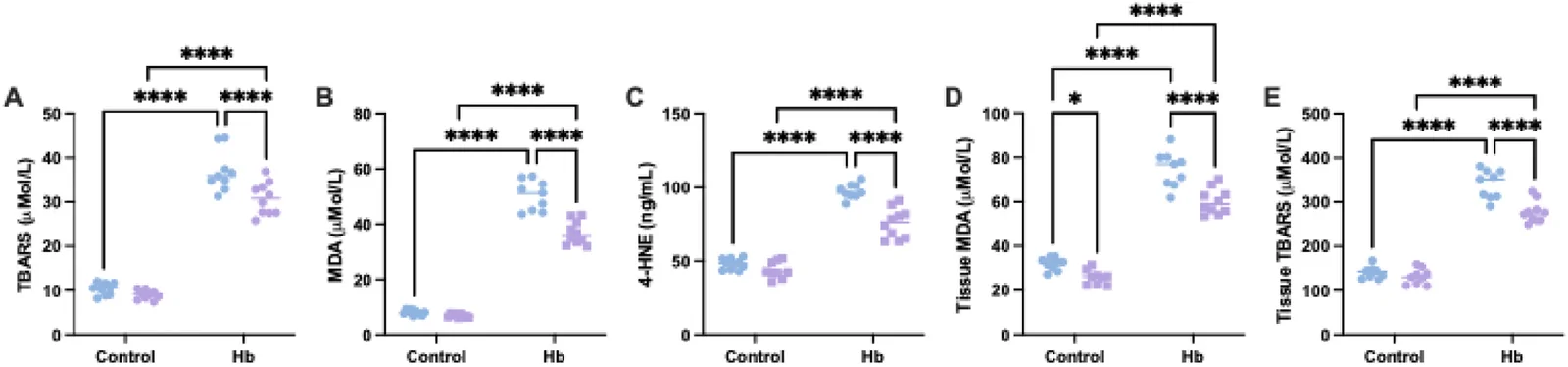
Quantification of lipid peroxidation in (A–C) Plasma malondialdehyde (MDA), thiobarbituric acid reactive substances (TBARS), and 4-hydroxynonenal (4-HNE) levels after six weeks of Hb infusion. All markers of lipid peroxidation were elevated in Hb-treated mice, with males exhibiting higher levels than females. (D–E) Renal MDA and TBARS concentrations showing a similar increase with Hb exposure, attenuated in females. Data is presented as individual values. Statistical significance between experimental groups is denoted by * (p < 0.05), **(p < 0.001), and *** (p < 0.0001).

To corroborate fluorescence-based ROS trends with quantitative biochemical endpoints, lipid peroxidation was assessed by measuring malondialdehyde (MDA), thiobarbituric acid reactive substances (TBARS), and 4-hydroxynonenal (4-HNE) in plasma and MDA/TBARS in renal tissues (Fig. 6). In plasma (Fig. 6A–C), chronic Hb infusion significantly elevated MDA, TBARS, and 4-HNE levels compared with sex-matched controls, indicating increased systemic lipid peroxidation. These elevations were most pronounced in male Hb-treated mice, while females exhibited significantly lower levels across all three plasma markers, suggesting enhanced antioxidant protection and reduced lipid oxidative injury. In renal tissues (Fig. 6D–E), Hb exposure similarly increased MDA and TBARS concentrations relative to controls, consistent with oxidative damage at the organ level. Again, female kidneys displayed markedly lower MDA/TBARS levels compared with male counterparts, supporting a sex-dependent attenuation of lipid peroxidation.

Overall, these findings confirm that chronic Hb infusion promotes widespread lipid oxidative damage, with male mice showing higher susceptibility to Hb-induced redox imbalance. The protective phenotype observed in females parallels their preserved mitochondrial coupling efficiency and respiratory reserve, indicating a resistance to oxidative stress–driven mitochondrial changes.

### 3.6. Systemic effects of Hb exposure

Moreover, although the heart and kidneys were the primary organs of interest, we also assessed systemic inflammatory responses to determine whether sex differences observed in target organs extended systemically (Supplemental Figure 2). Plasma levels of IL-6 and IL-10 were significantly elevated in both males and females compared to their controls, but no differences were observed between sexes, highlighting that sex-dependent effects can be organ-specific. Consistent with this, plasma BUN was significantly lower in females than in males, suggesting that systemic measures reflect organ performance and capture sex differences.

Finally, since the liver is responsible for processing most of the acellular Hb, liver enzymes were evaluated (Supplemental Figure 3). Females exhibited lower bilirubin and AST levels compared with males. However, both sexes showed significant elevations relative to their controls, indicating that chronic Hb exposure affects liver function in both sexes while still demonstrating sex-specific differences. These sex-specific differences may arise from hormonal regulation of heme oxygenase (HMOX1), a key enzyme in bilirubin metabolism, which is modulated by estrogen [30].

## 4. Discussion

This study demonstrates that chronic exposure to circulating Hb elicits cardiac and renal impairments with sex-specific differences. After six weeks of daily Hb injections, male animals exhibited significant declines in cardiac function, including reduced EF, FS, SV, and CO. In parallel, males demonstrated impaired renal function as reflected by reduced GFR, delayed clearance kinetics, and elevated biomarkers of tubular injury and inflammation. In contrast, females showed significantly lower impairment in both cardiac and renal function, exhibiting partial preservation of contractile and filtration capacity along with reduced injury and inflammation. These results support the idea that cell-free Hb is directly cytotoxic to many organs and that sex acts as a critical biological variable in modulating susceptibility to injury.

The cardiac dysfunction in males is consistent with prior evidence linking cell-free Hb to oxidative stress and impaired myocardial performance [43–46]. Hb extravasation into the myocardium has been shown to disrupt calcium handling and generate ROS, both of which compromise contractility [47–49]. However, the direct in vivo contribution of Hb to chronic cardiac functional decline had not been clearly demonstrated before this study. Our results align with preclinical studies of HBOCs, which have similarly reported impaired cardiac function and troponin elevation [50]. Notably, the significantly less impairment of cardiac function in females suggests that sex-specific protective mechanisms may mitigate Hb-induced cardiotoxicity

Renal findings further highlight sex disparities in response to chronic Hb. Male animals displayed reduced GFR and higher levels of creatinine, KIM-1, and urinary NGAL, consistent with tubular injury. Inflammatory profiling revealed elevated IL-6 in males, whereas females exhibited higher IL-10, suggesting that immune regulation may contribute to renal protection in females. These findings mirror clinical observations in sickle cell disease, where females experience slower progression of renal decline, and are consistent with reports in chronic kidney disease literature of improved outcomes in women [30,31,51].

Our data also demonstrate that chronic Hb exposure induces organ-specific mitochondrial respiration alterations, which likely explains the observed functional. Males exhibited significant reductions in basal and ATP-linked respiration and maximal respiratory capacity in both cardiac and renal tissues, indicating impaired oxidative phosphorylation and limited energetic reserve. This bioenergetic deficit may explain the impaired myocardial function and reduced filtration capacity observed in males, as both organs rely heavily on ATP to sustain contraction-relaxation coupling and tubular solute transport. Chronic Hb exposure may further exacerbate injury through heme- and iron-mediated oxidative stress, calcium dysregulation, and endothelial dysfunction, increasing cellular damage and inflammatory signaling [23,47,48], as shown by elevated IL-6 in males. Although fluorescence-based ROS assays provided qualitative trends, biochemical quantification of lipid peroxidation confirmed that chronic Hb exposure induces systemic oxidative injury. The correlation between increased lipid peroxidation and reduced mitochondrial reserve supports a mechanistic link between oxidative stress and bioenergetic impairment in males.

By contrast, females preserved mitochondrial bioenergetics despite showing higher proton leak, which may represent a protective response that limits ROS generation while maintaining ATP production. Higher mitochondrial reserve in females could enhance their capacity to tolerate oxidative and metabolic challenges, compared to males [52]. Moreover, lower lipid peroxidation levels (MDA/TBARS) were detected in females. Estrogens can enhance mitochondrial efficiency, scavenge ROS, increase NO bioavailability, and promote IL-10-mediated immune regulation [53–56], therefore contributing to the observed relative protection of cardiac performance and renal filtration. These results show possible sex-based mitochondrial adaptability as a central driver of chronic Hb-induced multi-organ injury and highlight sex-dependent bioenergetics as a key determinant of outcome.

These findings may be further supported by known baseline sex differences in mitochondrial function. Female controls generally exhibit higher intrinsic mitochondrial respiration in cardiac tissue, particularly during complex I and II activation, with less pronounced differences in renal tissues compared to males. Females also demonstrate greater proton leak and lower ADP sensitivity, which may reflect adaptive mechanisms to optimize oxygen utilization. In contrast, males typically exhibit higher mitochondrial ROS production and greater expression of oxidative phosphorylation genes [57]. Additionally, female cardiac muscle shows greater sensitivity of fatty acid oxidation to malonyl-CoA, favoring triglyceride incorporation. Together, these inherent differences suggest that female mitochondria may be better equipped to balance energy production and oxidative stress, whereas males may be more susceptible to ROS-mediated injury under chronic Hb exposure.

Beyond organ-specific effects, the convergence of functional and molecular data in this study supports the hypothesis that chronic Hb toxicity independently drives pathological changes. The ability of Hb to scavenge NO and release free heme provides a clear mechanism for both vascular dysfunction and direct cellular injury observed in this study. These findings have important implications for the development of bioengineered therapeutics, including HBOCs and targeted interventions for hemolytic anemias. Our results underscore why current HBOCs cannot be used chronically, as the downstream toxic effects of free Hb remain unmitigated. This highlights the urgent need for innovative engineering strategies to design safer HBOCs and develop molecules capable of scavenging circulating Hb, heme, and iron thereby reducing systemic toxicity.

The study findings reveal effects similar to those observed in clinical studies of patients with chronic hemolysis, in which sustained intravascular hemolysis leads to elevated circulating Hb, oxidative stress, and progressive cardiorenal injury. Like our model, human studies have shown correlations between plasma cell-free Hb levels, endothelial dysfunction, and reduced renal filtration rates [1,58,59]. However, unlike clinical conditions, this animal protocol isolates the direct effects of Hb exposure in the absence of anemia, chronic inflammation, or additional red blood cell components and fragments, allowing an understanding of Hb toxicity independent of confounding comorbidities. This highlights that many of the systemic complications observed in humans likely stem from cumulative Hb and heme toxicity, supporting the translational relevance of our findings while highlighting the limitations of extrapolation to disease contexts.

This study has several limitations. First, experiments were conducted in healthy rodents, which lack the chronic inflammatory and anemic background present in hemolytic disorders. This may underestimate or alter the pathophysiological impact of Hb in disease-relevant contexts. Second, only a single exposure duration (six weeks) was assessed, limiting the resolution of early versus late mechanisms of injury. Nonetheless, given our work with acute Hb, our group wanted to extend to a chronic model [60]. Third, while functional and biomarker data clearly demonstrated sex differences, this study did not directly include mechanistic pathways such as hormone signaling. Further studies will investigate different hormonal levels associated with changes seen due to possible hormonal protection, including the role of 17β-estradiol as suggested by the literature [61]. These limitations highlight important future directions, including the use of disease models, multiple time points, and mechanistic interventions to establish causal pathways and sex specific differences.

In conclusion, this study demonstrates that chronic Hb exposure impairs both cardiac and renal function regardless of biological sex. Nonetheless, multi-organ injuries in males seem more significant, while females are relatively protected. The combination of functional impairment, elevated injury biomarkers, and inflammatory signaling in males provides compelling evidence that free Hb acts as a driver of multi-organ toxicity under chronic conditions. Female protection may reflect the integration of anti-inflammatory, antioxidant, and hormonal mechanisms. These findings underscore the translational importance of considering sex differences in the pathophysiology of hemolytic disorders and point toward the potential for sex-specific therapeutic approaches to mitigate Hb-induced organ injury.

## Supplementary Material

suplementary

## Data Availability

Data will be made available on request.

## Appendix A. Supporting information

Supplementary data associated with this article can be found in the online version at doi:10.1016/j.biopha.2026.119439.
